# Cognitive training interventions for substance use disorders: what they really offer?

**DOI:** 10.3389/fpubh.2024.1388935

**Published:** 2024-04-17

**Authors:** Zahra Pazoki, Mohammad Taghi Kheirkhah, Shahriar Gharibzadeh

**Affiliations:** ^1^School of Behavioral Sciences and Mental Health, Iran University of Medical Science, Tehran, Iran; ^2^Institute for Cognitive and Brain Sciences, Shahid Beheshti University, Tehran, Iran

**Keywords:** cognitive training, substance use disorders, effectiveness, everyday life, precision medicine

## Abstract

Cognitive training (CT) has emerged as a potential therapeutic approach for substance use disorders (SUD), aiming to restore cognitive impairments and potentially improve treatment outcomes. However, despite promising findings, the effectiveness of CT in real-life applications and its impact on SUD symptoms has remained unclear. This perspective article critically examines the existing evidence on CT for SUD and explores the challenges and gaps in implementing CT interventions. It emphasizes the need for clarity in expectations and decision-making from a public health standpoint, advocating for comprehensive studies that consider a broader range of SUD consequences and utilize measures that reflect patients’ actual experiences.

## Introduction

Drug addiction, a leading risk factor for morbidity and mortality, presents significant challenges to global health. The treatment burden associated with this issue is substantial, and any efficient approach that can aid in its management is valuable. Neuropsychological interventions, including cognitive training (CT), are increasingly being recognized as effective methods for addressing cognitive impairments associated with substance use disorders (SUD) (1).

In recent decades, there has been increased attention to the cognitive aspect of drug addiction, both from the perspectives of the cognitive origins of substance use and the impact of substance use on cognitive functions (2). It is now widely recognized that cognitive impairments may occur as a result of SUD (3, 4). Moreover, pieces of research have demonstrated promising therapeutic effects on cognitive functions in patients with SUD (5).

CT interventions for SUD patients primarily take a restorative approach, utilizing repeated exercises. These interventions can be supplemented with compensatory strategies to improve working memory, executive functioning, and, in some cases, verbal learning, problem-solving, attention, and processing speed (6). It is believed that by improving higher order cognition, patients may gain better control over their consumption habits and become more actively engaged in their treatment process (7). The evidence indicating that individuals with SUD are at a heightened risk of cognitive impairments (8), and that cognitive impairments may predispose individuals to developing SUDs (9), underscores the importance of targeting cognitive impairments in intervention (10).

However, in some studies, the effectiveness of CT has been questioned, and evidence against being optimistic about it has been presented. For example, research has demonstrated that domain-specific training often fails to transfer to other cognitive functions (11) and has minimal impact on general cognition (12). Additionally, it has been suggested that the underlying mechanisms of CT suffer from a lack of clarity, and CT interventions may not necessarily target their intended objectives (13). Peckham (14) points out the reasons why CT may not be effective or transferable to everyday life. Accordingly, CT interventions fail to address individuals’ specific needs, do not align with the timing and context of symptoms experienced in real life, and overlook cultural and racial diversity.

Moreover, designing and developing interventions for SUD has always been challenging due to factors such as the diversity and complexity of SUDs (15), high relapse rates (16), high comorbidity (17), limited treatment availability (18), and individual differences in treatment response (19). These challenges, along with the limitations of CT interventions in transferring to real life, raise questions about what they offer.

This perspective article reviews the current understanding of CT within the context of SUD and raises questions regarding its impact on cognitive functions and substance use outcomes, as well as its potential applicability to real-life situations. Additionally, it emphasizes the significance of this issue for public health and outlines the way forward to achieve a more conclusive understanding.

## Cognitive training for substance use disorders

The primary argument for the importance of focusing on CT interventions for SUD is that, given the association between SUD and cognitive impairments (20), which may persist even after recovering from SUD, responding to conventional treatments can become more challenging (21). These remaining impairments can also heighten the risk of relapse, diminish the quality of life, and negatively impact social functioning (22). It is hypothesized that akin to other conditions involving cognitive impairment, enhancing executive functions may lead to increased engagement in treatment, consequently reducing substance consumption (23). Based on this argument, some claim that managing cognitive deficits is an important gap in therapeutic efforts for SUD (9). Some studies have gone further and explicitly suggest that CT interventions can lead to a reduction in SUD symptoms (24). However, the long-term effects of these types of interventions on the everyday life performance of patients are still in an aura of uncertainty (25).

While a systematic review of the literature was beyond the scope of the present study, a search was conducted in bibliometric databases for studies on CT for SUD. Previous reviews were also explored to identify additional potential references. Most existing studies have employed a restorative approach, focusing on enhancing working memory and executive functions. Some have incorporated compensatory strategies in conjunction with cognitive training. Others have investigated the impact of training on outcomes related to substance use (e.g., days of use and abstinence, severity of SUD symptoms, treatment duration, drug urinalysis, etc.) (6, 26, 27). Our current understanding of CT for SUD suggests that interventions are often effective on exercised tasks and may also have effects on general cognition. They may also affect the outcomes related to substance use in some cases. However, the findings are mixed and heterogeneous (5, 28). The impact of CT on craving, relapse, and other outcomes such as family and social status, and quality of life has not been addressed much. Additionally, there is insufficient evidence regarding the effect of CT on urine drug tests. The ability to transfer changes to everyday life cannot be determined based on the current state of the art. Furthermore, there is a lack of evidence regarding the influence of demographic diversity and other potential moderators on the impact of CT in SUD. Previous review articles have not been able to provide a conclusive decision on the effectiveness of CT on substance use outcomes or recommend a standard protocol regarding the type, procedure, and duration of intervention for clinical practice (5).

## Concerns regarding the empirical evidence

To reach a deeper understanding of what CT interventions truly offer us, it is essential to question them from various aspects. First, to what extent is it expected to observe effects on the primary outcomes (cognitive impairments) and what are the impacts on patients’ real-life cognition? Studies have demonstrated that CT can impact cognitive impairments, but there is limited evidence regarding its ability to transfer this effect to other cognitive domains (6, 29). A review conducted by Caballeria et al. identifies CT interventions with preliminary impacts on cognition and behavioral responses. However, it suggests that there is insufficient evidence to support their effectiveness in functional domains in real life (26). It must be noted that in the meantime, many published interventions have design flaws and shortcomings (i.e., small sample size, lack of result replication, low specificity of interventions, lack of assessor blinding, inadequate randomization procedures, failure to report dropouts, and moderate to high risk of bias) that prevent definitive conclusions regarding their effectiveness. Even though CT interventions may affect users’ cognitive performance on trained tasks, it is still unclear what effects this improvement will have on their cognitive performance in everyday life (27).

Second, to what extent can it be expected that CT interventions are effective on secondary outcomes (outcomes related to substance use)? The current evidence regarding the effect of CT on outcomes related to substance use includes mixed findings (28). In some studies, CT has demonstrated a reduction in consumption (30) and consumption-related problems (30), while in others, no difference was observed between the treatment and control groups (31, 32). Overall, the findings regarding the impact of CT on the severity of SUD symptoms and other substance use related outcomes are conflicting. It remains uncertain which changes can be expected from which CT protocol. Moreover, while certain cognitive impairments, like those in executive functions are often considered core to compulsive substance use, it is difficult to see how cognitive training and remediation could serve as treatments for SUD in and of themselves as is sometimes stated (1). Given the multifaceted and complex nature of addiction, it is still unclear how CT can affect various aspects of drug addiction, including its biological, psychological, and sociocultural determinants, and overcome their impacts in real-life settings.

Third, which mediator and moderator factors play an effective role in this relationship? For example, the role of individual differences in the relationship between CT and SUD has been less addressed. SUD is shown to have varying effects on different individuals (17). Lifestyle and health behaviors, such as nutritional habits, physical activity, and sleep routines, can significantly influence cognitive performance in everyday life (33, 34). It has also been observed that SUD and health behaviors are closely related (35–37). The effects that these variables can have on the association between CT and SUD are still unclear. Recent findings, however, underscore the significance of precision medicine for specific subgroups (38–41). In general, there is substantial heterogeneity among the findings of cognitive training trials in the population of patients with SUD (6).

## Public health concerns

The expanding market for CT solutions in various neurological and psychiatric conditions (42), raises concerns about prioritizing product development and customer service over evidence-based treatment. On the other hand, neglecting intervention shortcomings or overestimating their potential may lead to inappropriate allocation of resources. This, in turn, can lead to a less effective overall response to related health issues and take away the opportunity from other potentially effective approaches. Particularly, in the context of substance use, the impact of resource misallocation on ensuing public health outcomes is substantial (43). Accordingly, if the basic questions about the effectiveness of CT interventions for SUD are not answered, it may lead to misunderstanding by public opinion.

By conducting more rigorous research and fostering transparency in the development and evaluation of CT interventions for SUD, stakeholders can work toward improving outcomes for individuals struggling with substance use and promoting more effective public health responses to this issue.

## The way forward

Before a conclusion can be reached about the role of CT interventions in SUD, it is necessary to determine its limitations and to clarify the expectations that the health care service holds regarding its use. Given the case of our alignment with laboratory insights into improved cognitive performance, it remains to be determined whether this is something we can apply to patients in real-life settings. These interventions, in their optimal form, are intended to be used as adjunct treatments and do not offer a path toward alternative treatment for SUD. The benefits of these interventions across various populations are not clearly evidenced as well.

When implementing CT interventions, attention should be paid to factors that may mediate or moderate primary and secondary outcomes (i.e., cognitive performance and outcomes related to substance use either in laboratory or real-life settings). Individual differences should also be taken into account when evaluating cognitive functions and designing CT interventions. This can help explain the variability in treatment outcomes and allow for personalized interventions based on the patient’s specific needs. In this line, in the context of mental health and well-being, a recent meta-analysis using machine learning models has proposed moderators that can be used to determine which groups of people benefit most from which CT protocols (44). Conducting such studies in the field of substance use disorders could potentially yield significant benefits.

The lack of sufficient evidence of the impact of CT interventions on SUD, as much as it makes it difficult to reject the null hypothesis, also increases the doubts to confirm it. Conducting RCTs, field studies, and using registered datasets can contribute to a more definite conclusion of the effectiveness of CT on substance use in daily life. Needs assessment studies that address individual differences in the neuropsychopathology of SUD (e.g., genetic determinants, neurobiological systems, cognitive and personality profiles, ethnicity and culture, age, gender, habits, motivation, etc.) (19, 45) could also be helpful for this purpose. Additionally, if cognitive deficit management is believed to be a gap between the treatment of SUD and its effectiveness, we suggest that studies on the effectiveness of CT interventions consider the broader domains of the consequences of SUD (e.g., abstinence from drugs or alcohol, personal and social functioning, mental and physical health, and health risk behaviors) as secondary outcomes (46). Given the limited evidence of the effectiveness of CT interventions on cognitive performance and SUD symptom severity in real-life scenarios, it might be advantageous to employ measures that, along with self-reporting methods, reflect the desired outcomes in a manner close to patients’ actual experiences and away from deception (e.g., collateral Reports, clinician Ratings, implicit measures, etc.) (47, 48).

## Data availability statement

The original contributions presented in the study are included in the article/supplementary material, further inquiries can be directed to the corresponding author.

## Ethics statement

Ethical approval was not required for the study involving humans in accordance with the local legislation and institutional requirements. Written informed consent to participate in this study was not required from the participants or the participants’ legal guardians/next of kin in accordance with the national legislation and the institutional requirements.

## Author contributions

ZP: Conceptualization, Investigation, Writing – original draft. MK: Investigation, Writing – original draft, Writing – review & editing. SG: Supervision, Writing – review & editing.
